# Association of orthostatic hypertension identified according to different definitions with cardiovascular disease. The PARTAGE study

**DOI:** 10.1097/HJH.0000000000004177

**Published:** 2025-11-26

**Authors:** Paolo Palatini, Lucile Admant, Sylvie Gautier, Carlos Labat, Paolo Salvi, Davide Agnoletti, Athanase Benetos

**Affiliations:** a Department of Medicine - University of Padova, Padua, Italy; b INSERM DCAC University of Lorraine, Nancy; c Geriatric Department, CHRU de Nancy, University of Lorraine, Nancy, France; d Department of Cardiology, Istituto Auxologico Italiano, IRCCS, Milan; e Cardiovascular Internal Medicine, IRCCS AOU Sant’Orsola Hospital; f Cardiovascular Internal Medicine, Medical and Surgical Sciences Department, University of Bologna, Bologna, Italy

**Keywords:** blood pressure, cardiovascular, orthostatic, response, risk, standing

## Abstract

**Objective:**

Diagnostic criteria for an exaggerated BP increase in response to standing (ERTS) are still debated making it difficult to interpret data regarding the cardiovascular risk associated with ERTS. The aim of the present study was to identify the ERTS definition that was most strongly associated with cardiovascular disease.

**Design and methods:**

The study was conducted within the frame of the PARTAGE study, in 920 individuals aged 80 years or older. BP was measured 1 min and 3 min after standing up. Participants were classified into three groups according to whether they had normal response to standing (reference group), orthostatic hypotension, or ERTS defined using 16 different definitions. The risk of adverse cardiovascular outcomes was explored by means of multivariable survival Cox analyses.

**Results:**

ERTS was associated with both cardiovascular events and mortality when it was identified according to three definitions: SBP ≥20 mmHg in either orthostatic measurement [hazard ratios (HRs) (95% confidence interval, CI), 1.45 (1.03–2.03; *P* = 0.031)] and 1.71 (1.05–2.77; *P* = 0.030), respectively; SBP ≥15 mmHg in either orthostatic measurement ((1.43 (1.03–1.99; *P* = 0.032) and 1.82 (1.12–2.94; *P* = 0.015)), respectively; and SBP ≥20 mmHg and/or DBP ≥15 mmHg in either orthostatic measurement ((1.42 (1.03–1.98; *P* = 0.035) and 1.94 (1.19–3.17; *P* = 0.008)), respectively. The best model fit was found for SBP and DBP combined. No independent association with both outcomes was found for other ERTS definitions.

**Conclusions:**

The present results show that an SBP increase of >15–20 mmHg is a more important prognostic indicator than less pronounced increases of SBP. However, associating also an increase in DBP of ≥15 mmHg slightly increased the predictive value of ERTS.

## INTRODUCTION

In the last few years evidence has been accumulating that an exaggerated BP increase in response to standing (ERTS) is associated with an increased risk of mortality and adverse cardiovascular outcomes [1–5]. However, the lack of universally accepted definition of ERTS made it difficult to establish its actual clinical and prognostic significance. Most investigators used systolic BP (SBP) to define ERTS [1–9], a minority used diastolic BP (DBP) [10,11], and others a combination of the two [12–15]. Previous data suggest a different clinical impact of ERTS based on systolic or diastolic BP measurement [16] but only a few studies compared the prognostic value of the two pressures within the same population [11]. Although experts agree that SBP is the most important component from a clinical standpoint [3,17], especially in older subjects, DBP may also be of interest in understanding the pathophysiology and clinical significance of orthostatic BP changes [16,17]. Another controversial point is the identification of the optimal threshold level for both SBP and DBP responses to standing (RTS), as many different cut-offs have been used in the literature to define ERTS. In most studies a ≥20 mmHg increase was used to define ERTS for SBP [1–4,6,7] and a ≥10 mmHg increase for DBP [12,13], but also other cut-points were used all based on arbitrary criteria [5,8,10]. Improving the diagnostic testing of RTS and identifying the most appropriate definition of ERTS can be of help to prevent the harmful effects of hyperreactivity to standing. According to a recent Consensus document, both SBP and DBP orthostatic changes should be taken into consideration in clinical studies and different SBP and DBP RTS cut-offs should be tested [17]. The choice of the most appropriate diagnostic criteria should ideally be based on outcome data.

In a previous analysis of the predictive values of blood pressure and arterial stiffness in institutionalized very aged population (PARTAGE) study, we showed that ERTS was associated with higher cardiovascular morbidity and mortality in a population of people 80-year-old or older [6]. In that study a ≥20 mmHg increase in SBP was arbitrarily used to define ERTS and no other definition was tested. In view of the present controversy about the appropriate identification of the individuals with ERTS, we decided to reexamine the PARTAGE data, testing the association of major adverse cardiovascular events (MACE) and cardiovascular mortality with a variety of ERTS definitions based on SBP and/or DBP using different threshold levels for both pressures.

## METHODS

The PARTAGE is an observational cohort study of 1130 individuals aged 80 years or older [6,18]. Participants were recruited by 4 French (Nancy, Dijon, Paris, and Toulouse) and 2 Italian (Cesena and Verona) university hospital centers between January 2006 and June 2008. Participants were included if they were aged ≥80 years and were living in nursing homes. A total of 72 nursing homes participated in the PARTAGE in France and Italy. The rationale and design of the PARTAGE study have been described previously [18]. This age category was chosen because the association of BP levels and BP reaction to standing with cardiovascular disease in very elderly institutionalized persons with several comorbidities was a controversial issue, with several studies showing a lack of such a relationship. The present analysis was performed in 920 participants (706 women and 214 men) with a mean age ± SD, 87.6 ± 4.6 years. We considered the 931 participants who were able to assume the standing posture for at least 3 min, in whom two orthostatic BP measurements after 1 min and 3 min standing could be performed. In 11, there were one or more relevant missing variables and thus 920 participants were finally included for analysis. The sex distribution reflects the situation present in the nursing homes in France, according to the Ministry of Health data [19].

### BP measurements

SBP and DBP were measured at the brachial artery level using the Colson automated oscillometric device (Dupont Médical, Frouard, France). This device, marketed in France under the brand name Colson DM-H20, was validated according to the ESH protocol [20]. The mid-arm circumference was measured and the cuff width was chosen accordingly. BP measurements were performed in the sitting position by physicians or nurses after a 10-min rest. All measurements were repeated three times at 3-min intervals on the left arm according to the previously reported procedures [21]. For the 920 individuals who were able to reach and maintain the standing position, BP measurements were repeated at 1 and 3 min after standing. Orthostatic hypotension was defined as an SBP decrease of at least 20 mmHg and/or a DBP decrease of at least 10 mmHg at the first and/or the third minute of standing up in agreement with the American Autonomic Society and American Academy of Neurology guidelines [22]. This definition was maintained unchanged across the different regression models. Participants were classified into three groups according to whether they had normal response to standing (reference group), orthostatic hypotension, or ERTS.

### Definition of exaggerated blood pressure response to standing

**FIGURE 1:**
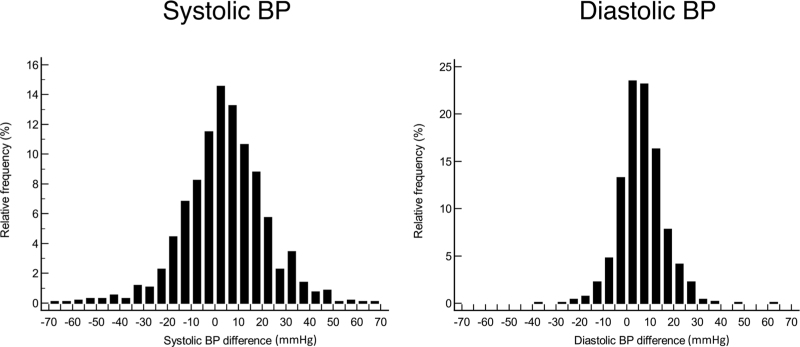
Plots of distribution of systolic and diastolic BP response to standing in 920 PARTAGE participants. BP indicates blood pressure.

In our previous analysis of orthostatic BP data in the PARTAGE study, ERTS was defined as an SBP increase of ≥20 mm Hg at the first and/or third minute of standing [6]. In the present study, 16 different definitions were tested using different combinations of SBP and DBP (Table 1). The 20 mmHg, 15 mmHg, and 10 mmHg cut-offs were used for SBP, and the 15 mmHg and 10 mmHg cut-offs for DBP. The SBP (10, 15, and 20 mmHg) and DBP (10 and 15 mmHg) cut-offs were chosen on the basis of previous investigations, because they were the threshold levels adopted in most outcome studies in the literature. The reason why the cut-offs ranged from 10 to 20 mmHg for SBP and from 10 to 15 mmHg for DBP lays on the different distribution of the SBP and DBP response to standing (RTS) in the PARTAGE. As shown in Fig. 1 and Figure S1 (Supplemental Digital Content), the RTS range was much narrower for DBP than for SBP. In particular, an RTS > 20 mmHg was observed in 14.6% of the participants for SBP, and only in 6.0% for DBP.

**TABLE 1:** ERTS definitions used as outcome predictors in the present study

**ERTS defined according to systolic BP**
• RTS ≥20 mmHg in either measurement
• RTS ≥20 mmHg in 1 min measurement
• RTS ≥20 mmHg in 3 min measurement
• RTS ≥20 mmHg in both measurements
• RTS ≥15 mmHg in either measurement
• RTS ≥15 mmHg in both measurements
• RTS ≥10 mmHg in either measurement
• RTS ≥10 mmHg in both measurements
**ERTS defined according to diastolic BP**
• RTS ≥15 mmHg in either measurement
• RTS ≥15 mmHg in both measurements
• RTS ≥10 mmHg in either measurement
• RTS ≥10 mmHg in both measurements
**ERTS defined according to systolic and diastolic BP combined**
• RTS SBP ≥20 or DBP ≥15 mmHg in either measurement
• RTS SBP ≥20 or DBP ≥10 mmHg in either measurement
• RTS SBP ≥20 and DBP ≥15 mmHg in either measurement
• RTS SBP ≥20 and DBP ≥10 mmHg in either measurement

ERTS, exaggerated blood pressure increase in response to standing; BP, blood pressure; DBP, diastolic blood pressure; RTS, response to standing; SBP, systolic blood pressure.

### Covariates

The collection of clinical data for the PARTAGE study has been described elsewhere [18,21]. All participants underwent a face-to-face interview, physical examination, anthropometry, and blood chemistry after an overnight fast. Biochemical tests were measured using standard methods. Covariates included in the survival analyses were age, sex, diabetes mellitus, and dyslipidemia. Comorbidities were quantified using the Charlson combined comorbidity index [23]. Functional status was assessed by the Katz Index of Independence in activities of daily living (ADL) [24]. The ADL scale assesses six primary and psychosocial functions: bathing, dressing, going to toilet, transferring, feeding, and continence. Subjects with low level of autonomy were not included in the PARTAGE (ADL score ≤ 2) [21], and thus subjects with ADL scores ranging from 3 to 6 were included in the survival analyses.

### Outcomes

Adverse outcomes were recorded every 3 months from inclusion to the end of the study, using a questionnaire addressed to the physicians at each nursing home [18,21]. In addition, two visits were conducted by the study investigators at the end of the first and second year of follow-up. For the present analysis the outcomes of interest were considered MACE and cardiovascular mortality. MACE included nonfatal cardiovascular events that led to hospitalization or a specific long-term new treatment as well as death from cardiac, cerebrovascular, and other vascular causes. Other details about the collection of adverse events were reported previously [25].

### Statistics

The total population (*n* = 920) was divided into three groups according to the orthostatic BP response. Orthostatic hypotension was diagnosed in 161 individuals (17.5%). The number of participants with ERTS and of those with normal BP response to standing (reference group) varied according to the definition of ERTS (Table 1). Continuous variables are presented as means (SD) and discrete variables as percentages. One-way ANOVA, followed by a Tukey test was used for comparisons of continuous variables and the *χ*^2^ tests for discrete variables. Agreement between the different ERTS definitions was assessed with kappa statistics, using the ≥20 mmHg SBP category as the reference definition. Weighted kappa (WK) was calculated according to the Cohen's method using linear weight [26]. The standard error and 95% confidence interval were calculated according to Fleiss *et al.* [27]. According to Altman, the strength of agreement can be defined as poor if kappa is <0.20, fair if kappa is 0.21–0.40, moderate if kappa is 0.41–0.60, good if kappa is 0.61–0.80, and very good if kappa is 0.81–1.00 [28]. The risk of MACE and cardiovascular mortality was explored by means of survival Cox analyses adjusting for several confounders and risk factors, including age, sex, sitting SBP and/or DBP, diabetes, smoking, dyslipidemia, ADL, and Charlson index. No violations to the proportional hazards assumption were detected by inspection of survival curves. Hazard ratios and corresponding two-sided 95% confidence intervals were derived from the regression coefficients in the Cox models. For all analyses, the follow-up time was defined as the period between the baseline visit and the last confirmed follow-up or date of event. The value of the –2Log likelihood was used to compare models in order to select the most informative model [29]. The model chi-square statistic was calculated, which is the difference of the values of the two log likelihood functions (i.e. the null model –2Log likelihood and the full model –2Log likelihood). All analyses were performed using Systat version 12 (SPAA Inc., Evanston, IL, USA) and Medcalc version 15.8 (MedCalc Software, Ostend, Belgium).

## RESULTS

The main demographic and clinical characteristics as well as the rates of MACE and cardiovascular mortality of the 920 participants stratified by sex are reported in Table 2. Women were the large majority of the PARTAGE population (76.7%), had a higher SBP (*P* = 0.007) and a lower Charlson index (*P* < 0.001) than men, and were less frequently current or past smokers (*P* < 0.001).

**TABLE 2:** Participants’ characteristics by sex

Variable	Men		Women	
	Mean	SD	Mean	SD
Age, years	87.3	4.8	87.6	4.5
Body mass index, kg/m^2^	25.2	4.1	25.7	4.6
Systolic blood pressure, mmHg	133.8	20.1	138.3	21.4
Diastolic blood pressure, mmHg	71.7	10.9	71.6	11.5
Heart rate, bpm	70.61	12.9	71.1	10.6
Activity of daily living index	5.1	0.9	5.1	0.9
Charlson index	6.3	2.0	5.7	1.7
Orthostatic SBP changes 1 min, mmHg	−3.1	20.0	0.5	18.7
Orthostatic DBP changes 1 min, mmHg	2.0	11.3	6.2	10.6
Orthostatic SBP changes 3 min, mmHg	4.3	17.6	9.2	17.8
Orthostatic DBP changes 3 min, mmHg	3.5	8.9	8.1	10.8

	Frequency	
Hypertension	57.5%	75.5%
Diabetes	15.0%	15.9%
Smoking	59.9%	11.2%
Dyslipidemia	21.0%	27.1%

DBP, diastolic blood pressure; SBP, indicates systolic blood pressure.

The RTS (orthostatic measurement – average of the three sitting measurements) was -0.3 ± 19.1 mmHg for SBP and 5.3 ± 11.0 mmHg for DBP at the 1-min standing measurement and increased to 8.1 ± 17.9 mmHg for SBP and 7.1 ± 10.6 mmHg for DBP at the 3-min measurement.

Plots of the distribution of SBP and DBP RTS (mean of 1 min and 3 min measurements) are shown in Figure 1. The RTS range was much greater for SBP than DBP, as confirmed by the box-and-whisker-plot (Figure S1, Supplemental Digital Content).

RTS of SBP, DBP and heart rate in the subjects divided according to whether they took (79.1%) or did not take (20.9%) antihypertensive therapy are shown in Figure S2 (Supplemental Digital Content). People on therapy had a slightly smaller response of DBP compared to those not on treatment while no significant differences were found for SBP and heart rate. However, the percentage of people on treatment was almost the same in the 3 RTS groups (Figure S3, Supplemental Digital Content).

**FIGURE 2:**
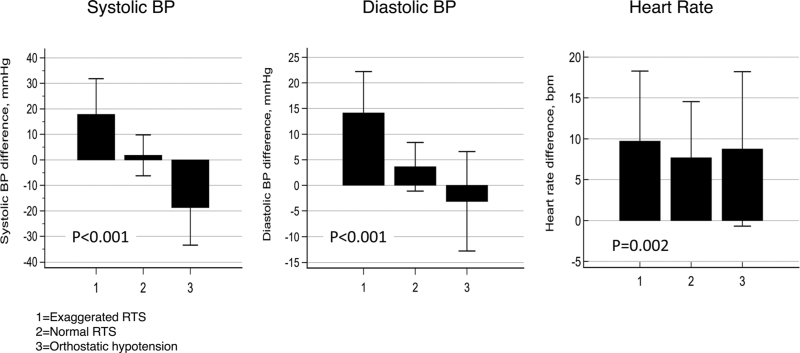
Systolic BP, diastolic BP, and heart rate response to standing in 920 PARTAGE participants stratified according to whether they had exaggerated RTS, normal RTS, or orthostatic hypotension. P-values from ANCOVA adjusted for age and sex. BP indicates blood pressure; RTS, response to standing.

Changes in SBP, DBP, and heart rate in the participants divided into three groups according to the orthostatic BP response are shown in Fig. 2. In the groups with ERTS and orthostatic hypotension heart rate response to standing was higher than in the participants with normal RTS. In particular, heart rate changes adjusted for age and sex were 9.7 ± 0.45 bpm (mean ± SEM) in people with ERTS and 7.7 ± 0.38 bpm in people with normal RTS (*P* = 0.001).

The prevalence of ERTS varied according to the definition and ranged from a minimum of 9.8% for ERTS defined as SBP ≥20 mmHg in both orthostatic measurements to a maximum of 48.5% for ERTS defined as SBP ≥20 mmHg and/or DBP ≥10 mmHg in either orthostatic measurement. The prevalence of orthostatic hypotension was 15.9% among the women and 22.9% among the men.

### Correlations

The Pearson's correlation coefficients between the RTS at 1-min and RTS at 3-min measurement were *R* = 0.74 and *R* = 0.55, respectively, for SBP and DBP. For both SBP (*R* = –0.22, *P* < 0.0001) and DBP (*R* = –0.20, *P* < 0.0001) there was an inverse correlation of the RTS with sitting BP, the higher the sitting BP the lower the RTS. SBP and DBP orthostatic changes were highly correlated with each other (*R* = 0.62, *P* < 0.0001).

### Prediction of cardiovascular outcomes

**FIGURE 3:**
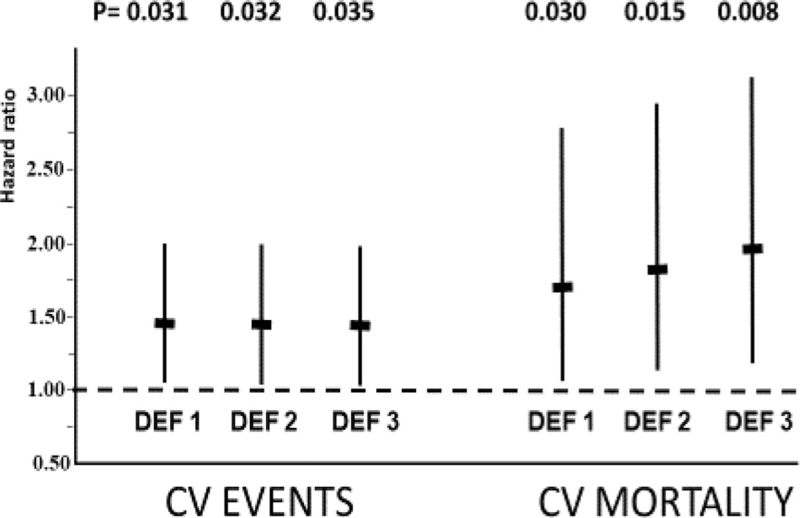
Risk of cardiovascular events and cardiovascular mortality for ERTS defined according to three different criteria in 920 PARTAGE participants followed for 2 years. Data from multivariable Cox models. Definition 1, SBP ≥20 mmHg; Definition 2, SBP ≥15 mmHg; Definition 3, SBP ≥20 mmHg, and/or DBP ≥15 mmHg. CV indicates cardiovascular; DEF, definition.

During a 2-year follow-up, 186 MACE (20.2%) and 90 cases of cardiovascular mortality (9.8%) were accrued. In the multivariable Cox analyses, ERTS was associated with both MACE and cardiovascular mortality when it was identified according to three definitions (Fig. 3): SBP ≥ 20 mmHg in either orthostatic measurement ((HRs (95% CI), 1.45 (1.03–2.03; *P* = 0.031)) and 1.71 (1.05–2.77; *P* = 0.030), respectively; SBP ≥15 mmHg in either orthostatic measurement (HRs, 1.43 (1.03–1.99; *P* = 0.032) and 1.82 (1.12–2.94; *P* = 0.015), respectively; and SBP ≥20 mmHg and/or DBP ≥15 mmHg in either orthostatic measurement (HRs, 1.42 (1.03–1.98; *P* = 0.035) and 1.94 (1.19–3.17; *P* = 0.008), respectively (Fig. 3). The prevalence of ERTS according to these three definitions was 25.0%, 34.6%, and 35.3%, respectively. For the prediction of MACE, a similar overall model fit was found for the three ERTS definitions (Table 3) whereas for the prediction of cardiovascular mortality, a slightly better model fit was found for the SBP ≥20 mmHg and/or DBP ≥15 mmHg definition (Table 3). According to kappa statistics a very good agreement was found between the above three ERTS definitions. The kappa coefficient was 0.87 for the SBP ≥20 mmHg in either orthostatic measurement definition versus the SBP ≥15 mmHg in either orthostatic measurement definition, and was 0.86 for the SBP ≥ 20 mmHg in either orthostatic measurement definition versus SBP ≥20 mmHg and/or DBP ≥15 mmHg in either orthostatic measurement definition.

**TABLE 3:** Overall regression model fit for three ERTS definitions

Outcome: all cardiovascular events	
**Definition 1**: SBP ≥ 20 mmHg in either orthostatic measurement	
**Model fit**	
Null model −2Log likelihood	2446.629
Full model −2Log likelihood	2417.941
Chi-squared	28.688
DF	10
Significance level	*P* = 0.0014

**Definition 2:** SBP ≥15 mmHg in either orthostatic measurement	
**Model fit**	
Null model −2Log likelihood	2446.629
Full model −2Log likelihood	2417.894
Chi-squared	28.735
DF	10
Significance level	*P* = 0.0014

**Definition 3:** SBP ≥20 mmHg and/or DBP ≥15 mmHg in either orthostatic measurement	
**Model fit**	
Null model −2Log likelihood	2446.629
Full model −2Log likelihood	2418.596
Chi-squared	28.033
DF	10
Significance level	*P* = 0.0018

Outcome: cardiovascular mortality	
**Definition 1**: SBP ≥20 mmHg in either orthostatic measurement	

**Model fit**	
Null model −2Log likelihood	1197.627
Full model −2Log likelihood	1169.114
Chi-squared	28.513
DF	10
Significance level	*P* = 0.0015

**Definition 2:** SBP ≥15 mmHg in either orthostatic measurement	
**Model Fit**	
Null model −2Log likelihood	1197.627
Full model −2Log likelihood	1167.993
Chi-squared	29.634
DF	10
Significance level	*P* = 0.0010

**Definition 3:** SBP ≥20 mmHg and/or DBP ≥15 mmHg in either orthostatic measurement	
**Model fit**	
Null model −2Log likelihood	1197.627
Full model −2Log likelihood	1166.409
Chi-squared	31.218
DF	10
Significance level	*P* = 0.0005

ERTS, exaggerated blood pressure increase in response to standing; DBP, diastolic blood pressure; SBP, systolic blood pressure.

An association with cardiovascular death but not with MACE was found for four ERTS definitions: SBP ≥20 mmHg at 1-min measurement (*P* = 0.029), DBP ≥10 mmHg at both orthostatic measurements (*P* = 0.011), SBP ≥ 20 mmHg and DBP ≥10 mmHg in either orthostatic measurement (*P* = 0.028), and SBP ≥20 mmHg and/or DBP ≥10 mmHg in either orthostatic measurement (*P* = 0.040). No independent association with either outcome was found for the other ERTS definitions.

In all the above survival analyses, orthostatic hypotension showed an increased risk of adverse cardiovascular outcomes than people with normal RTS, without a significant difference between orthostatic hypotension and ERTS (data not shown).

## DISCUSSION

The present results confirm that ERTS may be associated with unfavorable cardiovascular outcomes in the elderly as shown in a previous analysis of the PARTAGE study population [6]. In addition, they show that this association was present for three different ERTS definitions and that the best predictive value for cardiovascular mortality was provided by a combination of SBP and DBP RTSs.

In the last few years several lines of research have suggested a link between BP hyperreactivity to standing and risk of adverse cardiovascular outcomes [1–9]. This is why the latest ESH Guidelines have recognized the clinical value of standing BP measurement in the assessment of the hypertensive patient [30]. The ERTS-cardiovascular outcome association has been found to be present in middle-age people [5,31] as well as in elderly subjects [4,6–8,12] in general populations [6,7] and hypertensive cohorts [4,5,8,12]. However, the wide heterogeneity in the definition and diagnostic criteria poses a significant challenge on the actual clinical and prognostic significance of ERTS. In fact, inconsistent data have been reported in the literature especially for the association of ERTS with hard end-points. Although the majority of studies found a positive association between ERTS or orthostatic hypertension with cardiovascular events or mortality [4–8,12,13], some others did not observe any relationship [11,14]. In the CARDIPP study, even a lower risk of cardiovascular events was found in the participants with orthostatic hypertension [11]. In that study ERTS was defined as a rise of DBP ≥10 mmHg. However, the large majority of previous investigations have emphasized the role of SBP in evaluating the clinical significance of BP reactivity to standing, and recently some scientific societies defined ERTS as an orthostatic SBP increase ≥20 mmHg [3,17], a definition that was endorsed by the 2023 ESH recommendations for the management of arterial hypertension [30]. However, this definition was arbitrary and even the authors of a recent consensus document recognized that both SBP and DBP orthostatic changes should be taken into consideration in future clinical studies testing multiple cut-offs, and suggested that the optimal definition of ERTS should be based on outcome data [17].

In the present study, we tested the association of ERTS with MACE and cardiovascular mortality using 16 different definitions. These were based on SBP only, DBP only, or a combination of SBP and DBP. For both pressures, different cut-offs were used. The cut-off ranged from 10 to 20 mmHg for SBP and from 10 to 15 mmHg for DBP because of the narrower distribution of the DBP RTS. In addition, we tested the predictive capacity of ERTS being present in one or both orthostatic measurements.

Although the present results confirm the validity of the ≥20 mmHg SBP RTS definition, they show that also a lower SBP cutoff and a combination of the SBP and DBP RTSs have a similar prognostic accuracy. On the other hand, these three definitions showed very good agreement according to kappa statistics. In particular, it is noteworthy to observe that by combining the ≥ 20 mmHg SBP criterion with the ≥15 mmHg DBP criterion in the survival analysis the goodness of fit of the mortality model was improved compared to the SBP only definitions providing a more informative model. According to the SBP/DBP definition, the risk of cardiovascular mortality in our participants was almost doubled compared to those with normal RTS. This suggests that also the RTS of DBP should be taken into account when assessing the orthostatic BP changes also in very old individuals. Using SBP and DBP reactivities combined, ERTS was detected in over one third of the population allowing us to identify a larger number of participants at increased risk of cardiovascular events.

The precise mechanisms of orthostatic hypertension remain unclear. The observation that plasma norepinephrine increases more with standing in subjects with ERTS than those with normal RTS suggests that a major mechanism is an overshooting of sympathetic activation through the cardiopulmonary and/or arterial baroreceptor reflex and alpha-adrenergic vascular hyper-reactivity [1–4]. Small artery remodeling with endothelial dysfunction could contribute to the development of orthostatic hypertension in some individuals favoring an increase in vascular resistance on standing. While neurohumoral mechanisms appear to be crucial in mediating ERTS, as suggested also by the greater increase in heart rate found in the present study in the participants with ERTS, increased arterial stiffness can also contribute to the development of orthostatic hypertension (so-called vascular stiffness type) [1–3]. Data from Kario *et al.* have shown that augmentation index measured on standing was significantly greater in people with ERTS compared to people with normal RTS, whereas no significant difference was found in the augmentation index or pulse wave velocity measured in the supine position, indicating that an increase in reflection waves from the peripheral arteries on standing contributes to ERTS [32]. Thus, the pathophysiological backgrounds of orthostatic hypertension may differ in different patient groups with differential effects on the orthostatic response of SBP and DBP. A definition based on both SBP and DBP would include people with ERTS due to different pathogenetic mechanisms which may account for its better prediction of cardiovascular mortality.

### Limitations

Some limitations of our study should be mentioned. The present data were obtained in very old individuals, mostly women, with 80% receiving antihypertensive treatment and 50% having a history of cardiovascular complications [21] making the results of this study not generalizable to younger individuals and people with different clinical characteristics. In addition, although we used a large number of ERTS definitions, we cannot exclude that other SBP/DBP combinations with different BP cut-offs not explored in the present study could provide good prognostic accuracy.

## CONCLUSIONS

Our data obtained from very old subjects confirm that ERST can be found in a large portion of the general population spanning from one fourth to one third of people according to its definition and show that orthostatic hypertension is more common than orthostatic hypotension even in this age range. In addition, the present findings confirm that an SBP increase of more than 15–20 mmHg is a more important prognostic indicator than less pronounced increases of SBP or any isolated increase in DBP. However, a one-size-fits-all approach to the diagnosis of ERTS is probably inappropriate, considering the mechanistic complexity and heterogeneity of RTS in different clinical conditions and age groups [1,2,9]. In our octogenarian participants, associating also an increase in DBP of ≥15 mmHg slightly increased the predictive value of ERTS for cardiovascular death. As it is possible that the diagnosis of ERTS may vary across different subgroups, until more evidence is available to advance the present knowledge and better guide our practice, we recommend that several criteria be tested to assess BP reactivity to standing including both SBP and DBP orthostatic changes.

## ACKNOWLEDGEMENTS

None.

Funding: No funds were received for the present study.

### Conflicts of interest

There are no conflicts of interest.

## Supplementary Material

Supplemental Digital Content
