# Supplementary material for: Association of orthostatic hypertension identified according to different definitions with cardiovascular disease. The PARTAGE study
Source: J Hypertens. 2025 Nov 26;44(2):305–12. doi: 10.1097/HJH.0000000000004177 (PMC12746773; doi:10.1097/HJH.0000000000004177)
Supplement: Supplemental Digital Content [file jhype-44-305-s001.docx]

**ASSOCIATION OF ORTHOSTATIC HYPERTENSION IDENTIFIED ACCORDING TO DIFFERENT DEFINITIONS WITH CARDIOVASCULAR DISEASE. THE PARTAGE STUDY.**

Paolo Palatini (1), Lucile Admant (2), Sylvie Gautier (3), Carlos Labat (2), Paolo Salvi (4), Davide Agnoletti (5,6), Athanase Benetos (2,3)

(1) Department of Medicine - University of Padova, Padua, Italy

(2) INSERM DCAC University of Lorraine, Nancy France

(3) Geriatric Department, CHRU de Nancy, University of Lorraine

(4) Department of Cardiology, Istituto Auxologico Italiano, IRCCS, Milan, Italy

(5) Cardiovascular Internal Medicine, IRCCS AOU Sant’Orsola Hospital, Bologna, Italy.

(6) Cardiovascular Internal Medicine, Medical and Surgical Sciences Department, University of Bologna, Italy.

Figure S1. Systolic and diastolic BP response to standing in 920 PARTAGE participants. The box-and whisker plots show the median and the 25th and 75th percentiles; the whiskers indicate the 5th and 95th percentiles; estimates > 1.5 times the interquartile distance (i.e., outliers) are represented as single triangles.


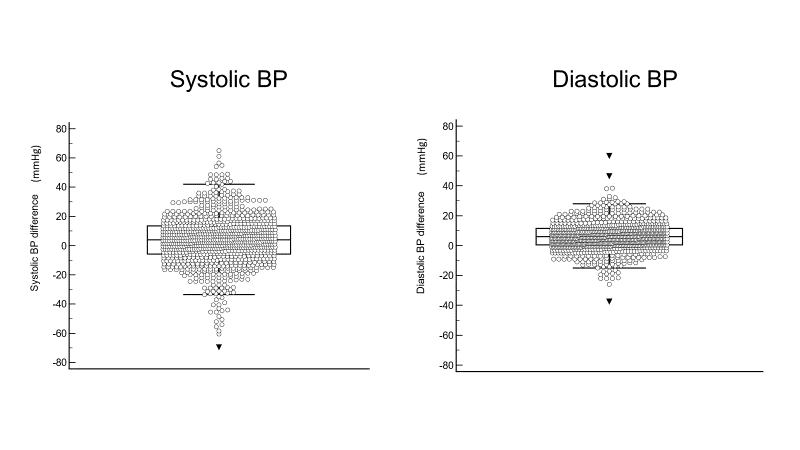


BP indicates blood pressure.

Figure S2. Systolic BP, diastolic BP, and heart rate response to standing in 920 PARTAGE participants stratified according to whether they received or did not receive antihypertensive therapy.


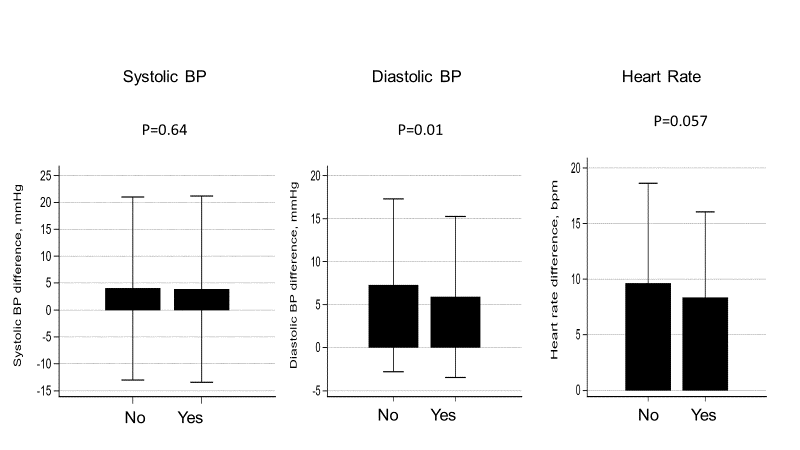


BP indicates blood pressure.

Figure S3. Rate of antihypertensive therapy in 920 PARTAGE participants stratified according to their blood pressure response to standing


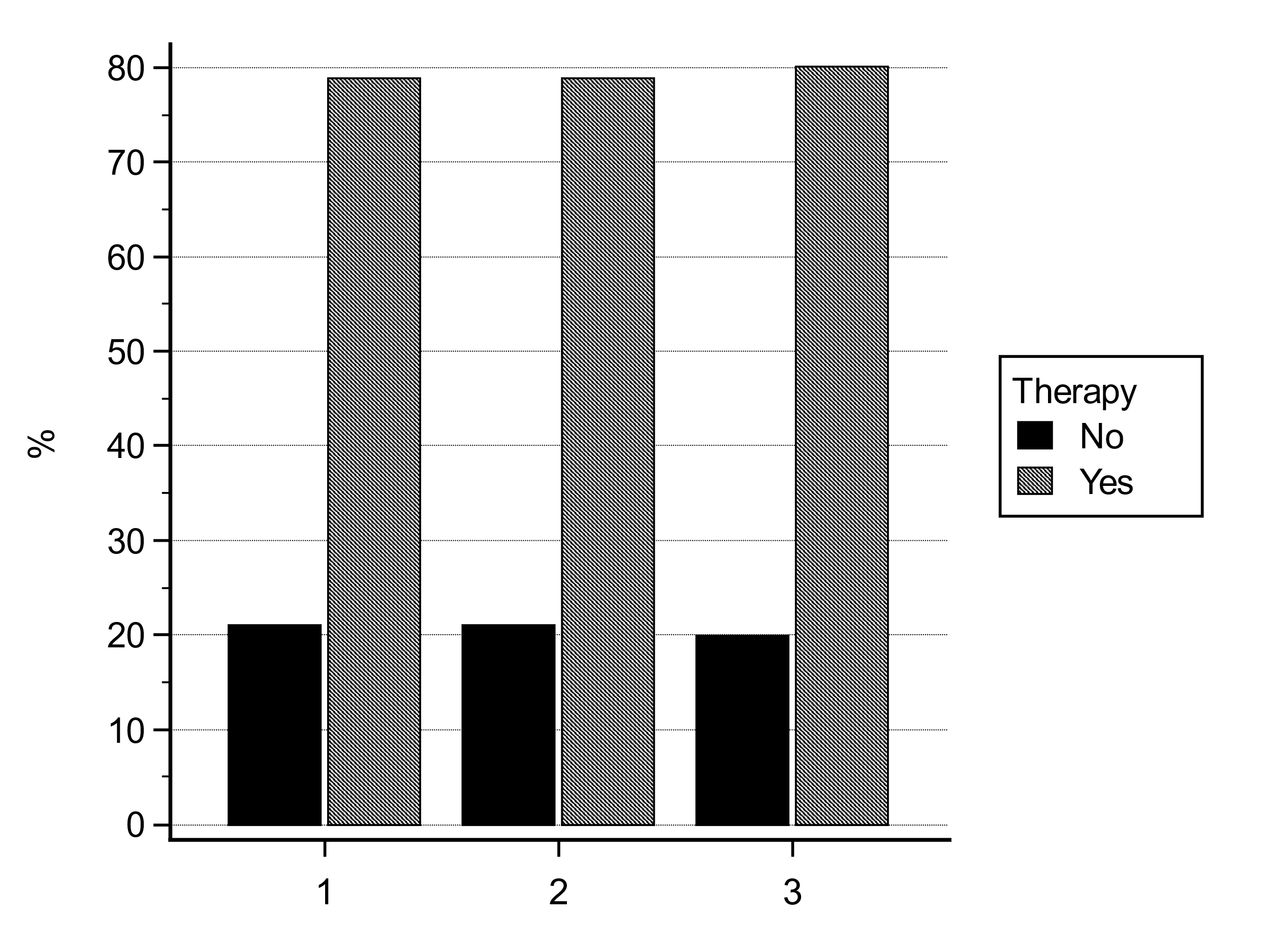


Normal

RTS

Exaggerated

RTS

Orthostatic

hypotension

RTS indicates response to standing.
